# Effects of preoperative plasma exchange therapy with albumin replacement fluid on blood coagulation in patients undergoing ABO-incompatible living-donor kidney transplantation using rotational thromboelastometry

**DOI:** 10.1186/s12871-018-0536-2

**Published:** 2018-06-19

**Authors:** Kazuhiro Shirozu, Naoyuki Fujimura, Yuji Karashima, Mizuko Ikeda, Hidehisa Kitada, Yasuhiro Okabe, Kei Kurihara, Tomoko Henzan, Sumio Hoka

**Affiliations:** 1 Department of Anesthesiology and Critical Care Medicine, Kyushu University Hospital, 3-1-1 Maidashi, Higashi-ku, Fukuoka, 812-8582 Japan; 2 Department of Anesthesiology, St. Mary’s Hospital, Kurume, Japan; 3 Department of Anesthesiology and Critical Care Medicine, Graduate School of Medical Sciences, Kyushu University, Fukuoka, Japan; 4 Department of Surgery and Oncology, Graduate School of Medical Sciences, Kyushu University, Fukuoka, Japan; 5 Center for Cellular and Molecular Medicine, Kyushu University Hospital, Fukuoka, Japan

**Keywords:** ROTEM, Thromboelastometry, ABO-incompatible living-donor kidney transplantation, Plasma exchange therapy with albumin replacement fluid

## Abstract

**Background:**

ABO-incompatible living-donor kidney transplantation (LDKT) requires immunotherapy and plasma exchange therapy (PEX). PEX with albumin replacement fluid reportedly decreases fibrinogen levels. However, no reports have described the effects of PEX with albumin replacement fluid on blood coagulation parameters and blood loss during the perioperative period. Therefore, we investigated the effects of preoperative PEX on blood coagulation parameters and blood loss during the perioperative period in patients undergoing ABO-incompatible LDKT as measured by rotational thromboelastometry (ROTEM®).

**Methods:**

Twenty-eight patients undergoing LDKT were divided into the PEX group (ABO incompatible with PEX, *n* = 13) and non-PEX group (ABO compatible without PEX, *n* = 15). ROTEM® parameters, standard laboratory test parameters, bleeding volume, and transfusion volume were compared between PEX and non-PEX group. MCE_platelet_, which represents platelet contribution to clot strength and where “MCE” stands for maximum clot elasticity, was calculated from the difference in MCE between EXTEM and FIBTEM.

**Results:**

The bleeding volume during surgery and the intensive care unit (ICU) stay was significantly higher in the PEX than non-PEX group (*p* < 0.01). Maximum clot firmness (MCF) of EXTEM (MCF_EXTEM_), MCF_FIBTEM_, and MCE_platelet_ was significantly lower in the PEX than non-PEX group (*p* < 0.01). In the PEX group, the bleeding volume during surgery was very strongly correlated with the baseline MCF_EXTEM_ and MCE_platelet_, and the bleeding volume during the ICU stay was strongly correlated with the postoperative MCF_EXTEM_ and MCE_platelet_.

**Conclusions:**

These results suggest that the increased blood loss in the PEX group during surgery and the ICU stay was associated with decreased platelet contribution to clot strength as measured by ROTEM®.

**Trial registration:**

UMIN-Clinical Trial Registry UMIN000018355. Registered 21 July 2015.

**Electronic supplementary material:**

The online version of this article (10.1186/s12871-018-0536-2) contains supplementary material, which is available to authorized users.

## Background

Living-donor kidney transplantation (LDKT) is a kidney replacement therapy performed to treat various end-stage kidney diseases. The performance of ABO-incompatible LDKT has recently increased because of the long waiting times for deceased-donor kidney transplantation. Plasma exchange therapy (PEX) is performed to prevent hyperacute rejection across the ABO antibody barrier before surgery [1]. PEX removes plasma proteins from the circulation, and the recipient plasma is replaced by albumin, fresh-frozen plasma (FFP), or a combination of both. To minimize the risk of viral transmission and/or anaphylactic reaction, 5% albumin is widely used as a replacement fluid during PEX [2]. Because coagulation factors are removed, the risk of coagulopathy is increased after PEX, especially when albumin replacement fluid is used. PEX with albumin replacement fluid reportedly leads to prolongation of activated partial thromboplastin time and prothrombin time, and to increasing international normalized ration [3]. Fluid management during renal transplantation mostly involves maintaining a sufficient intravascular volume and renal perfusion pressure. Large amount volume management is therefore recommended to ensure adequate kidney perfusion and stimulate urine production [4–6]. However, this fluid management regimen results in hemodilution, which might accelerate coagulopathy in patients undergoing ABO-incompatible LDKT with PEX performed with albumin replacement fluid [7]. Nonetheless, the coagulation changes that occur during ABO-incompatible LDKT with PEX performed using albumin replacement fluid remain unknown.

Rotational thromboelastometry (ROTEM®; TEM International GmbH, Munich, Germany) enables point-of-care coagulation monitoring device of viscoelastic clot strength in whole blood. ROTEM® was recently introduced to guide the transfusion of hemostatic blood components in the operating room, thus reducing blood transfusion and associated hospitalization costs [8–11]. Tholking et al. [3]. reported that PEX significantly altered the ROTEM® data regarding dilutional changes in coagulation parameters. However, they did not show whether these changes were associated with increased blood loss or caused increased rates of transfusion. Thus, the purpose of this study was to investigate the association of routine laboratory test parameters and ROTEM® variables with blood loss during and after ABO-incompatible LDKT with PEX performed using albumin replacement fluid.

## Methods

### Ethical considerations

The study protocol was approved by the institutional clinical research ethics committee (IRB: Clinical Research number #26–286, Kyushu University, Fukuoka, Japan) and registered at UMIN-CTR (UMIN000018355). This study complied with the declaration of Helsinki (2013).

This was an observational study of patients who underwent LDKT at our university hospital from October 2014 to March 2015. Informed consent was obtained from all participants included in this study. Patients with blood diseases and those undergoing anticoagulant and/or antiplatelet therapy were excluded. As a result, 28 patients were included in this analysis. The patients were divided into two groups: ABO compatible group without PEX (non-PEX group) and ABO incompatible group with albumin (PEX group). Between both group, some clinical examinations were compared.

### PEX

According to the standard protocol of our center, a venous dialysis catheter was inserted into the arteriovenous shunt or cubital vein prior to the first PEX session. PEX was performed using a membrane plasma separator (Plasmacure™ PE; Kawasumi Laboratories, Tokyo, Japan). All patients were treated with 1.5 to 2.0 L of 5% albumin (0.4–1.0 plasma volume) per session at an interval of 1 to 2 days. The last PEX session was uniformly performed the day before surgery in all patients. However, for patients whose fibrinogen levels were less than 180 to 200 mg/dl before PEX, FFP in addition to 5% albumin was used (Additional file 1). The number of PEX treatments required in the PEX group depended on the antibody levels (titer of < 1:32).

### Procedures

Anesthesia was induced by the intravenous administration of propofol and fentanyl. Rocuronium was administered to facilitate tracheal intubation. Anesthesia was maintained with isoflurane (1.0–1.5%) in an air/oxygen mixture with continuous remifentanil infusion and intermittent bolus infusion of fentanyl and rocuronium. Each patient’s electrocardiogram, oxygen saturation, and invasive arterial pressure and central venous pressure were monitored intraoperatively. Postoperative analgesia involved intravenous fentanyl infusion and infiltration of local anesthetics into the surgical sites.

Normal saline and human albumin 5% were infused to maintain the CVP at target 15 mmHg. RBC was infused to maintain the hemoglobin concentration above 7 g/dL. We administered FFP during surgery when preoperative plasm fibrinogen level was below 200 mg/dL. To maintain the CVP, 5% albumin and FFP were mainly administered in the non-PEX or PEX group, respectively.

### ROTEM® measurement data

Thromboelastometric measurements were performed on the quad-channel ROTEM Coagulation Analyzer. The results of ROTEM® were obtained from EXTEM and FIBTEM before surgery (baseline) and just after surgery in 13 patients in the PEX group and 15 patients in the non-PEX group. The run time of ROTEM® analysis was 60 min. Specifically, the maximum clot firmness (MCF), clot formation time, alpha angle, and clotting time of EXTEM and the MCF of FIBTEM were measured. EXTEM is regarded as the extrinsic coagulation system. For FIBTEM, cytochalasin D is added to inhibit conformational changes of platelet glycoprotein IIb/IIIa receptors [12]. Thus, fibrin polymerization can be specifically evaluated in the absence of attachment to platelets, and the clot strength based on fibrinogen alone can be evaluated [12, 13].

The “platelet component” of clot strength is expressed as the difference in clot strength between EXTEM and FIBTEM, as previously reported for platelet IIb/IIIa inhibitors [13–16]. and is calculated as follows (where “MCE” stands for maximum clot elasticity): MCE_platelet_ = MCE_EXTEM_ − MCE_FIBTEM_. The MCE was calculated as follows: MCE = (MCF *100) / (100 − MCF) [14].

### Hemostasis parameters and infusion measurement

Fibrinogen and platelets were compared between the two groups before surgery (baseline) and just after surgery. The prothrombin time-international normalized ratio (PT-INR) and activated partial thromboplastin time (APTT) were also compared between the two groups before surgery (baseline) and after surgery.

The amounts of perioperative fluid, including red blood cells, FFP, and 5% albumin, were also compared between the two groups.

### Statistical analysis

Power analysis (α = 0.05, β = 0.20) indicated that a subject sample size was sufficient (actual power: 0.95) for detecting a significant difference in bleeding during or after surgery between the PEX and non-PEX groups, using data collected in a post-study analysis. F test was performed to check whether comparing data in this study were normally distributed. Data are presented as mean ± standard deviation except for transfusion or bleeding data, which are presented as median [interquartile range] or ratio (Tables 1 and 2). Sidak’s multiple-comparison post hoc test was utilized for two-way analysis of variance. An unpaired *t*-test or unpaired *t*-test with Welch’s correction was used to detect differences in basic characteristics, clotting factors, and volume balance within groups. The relationship between ROTEM variables and the amount of bleeding during the perioperative period were determined using Pearson correlation coefficients within groups. Interpretation of size of correlation coefficient was defined as very strong: 0.9–1.0, strong: 0.7–0.9, moderate: 0.5–0.7, weak: 0.3–0.5, negligible: 0–0.3 [17].

**Table 1:** Basic demographic and clinical characteristics and coagulation factors of patients before PEX therapy

	PEX (+)	PEX (−)	*P* value
Number of patients	13	15	
Age (year)	42.2 ± 13.9	45.9 ± 14.9	0.496
Weight (kg)	59.4 ± 12.5	66.0 ± 15.7	0.239
Body mass index^a^	22.5 ± 4.0	23.5 ± 5.3	0.550
Anesthesia time (min)	415 ± 69.5	400 ± 87.2	0.601
Surgical time (min)	307 ± 55.9	299 ± 88.0	0.763
Male/female	6/7	10/5	0.445
Dialysis (%)^a^	53	40	0.705
Hypertension (%)^a^	92	87	1.000
Diabetic mellitus (%)^a^	23	40	0.435
Platelet (× 10^3^/μl)	205.2 ± 61.3	233.8 ± 57.2	0.213
Fibrinogen (mg/dl)	322.8 ± 87.4	299.8 ± 65.8	0.496
Hemoglobin (g/dl)	11.7 ± 1.6	11.7 ± 1.6	0.995
Hematocrit (%)	35.1 ± 5.3	36.0 ± 4.8	0.647

Data are presented as the ratio (%) and mean ± standard deviation. The two groups were compared using an unpaired t-test, unpaired t-test with Welch’s correction or ^a^Fisher’s exact test

*Abbreviations*: *% dialysis* percentage of dialysis, *BMI* body mass index (weight (kg) / height squared (m^2^))

**Table 2:** Comparison of coagulation factors between baseline and after surgery

	PEX (+)	PEX (−)	*P* value
APTT at baseline (sec)	30.0 ± 3.2	27.2 ± 2.4	0.170
APTT postoperatively (sec)	32.6 ± 5.2	31.5 ± 5.4	> 0.995
PT-INR at baseline (sec)	1.0 ± 0.1	0.99 ± 0.04	0.50
PT-INR postoperatively (sec)	1.0 ± 0.1	1.08 ± 0.06	0.040
Platelet at baseline (× 10^3^/μl)	181.6 ± 57.7	236.0 ± 59.2	0.025
Platelet postoperatively (× 10^3^/μl)	154.6 ± 45.0	215.2 ± 58.2	0.012
Fibrinogen at baseline (mg/dl)	154.4 ± 23.8	299.8 ± 65.8	< 0.001
Fibrinogen postoperatively (mg/dl)	175.0 ± 30.3	235.1 ± 45.8	0.002

Data are presented as mean ± standard deviation. Sidak’s multiple-comparison post hoc test was utilized for two-way analysis of variance

The Mann–Whitney test or Fisher’s exact test was used to compare the basic parameters between the two groups. All statistical analyses were performed using Prism 6 software (GraphPad Software, La Jolla, CA, USA), with *p*-values of < 0.05 considered statistically significant.

## Results

### Patient characteristics

Twenty-eight patients were enrolled in this study. Thirteen ABO-incompatible patients (PEX group) required preoperative PEX to remove anti-A or -B antibodies. The patient characteristics are shown in Table 1. There were no significant differences in the platelet count, fibrinogen level, hemoglobin concentration, or hematocrit between the two groups (Table 1).

### Changes in laboratory test parameters

Laboratory tests were performed before surgery (baseline) and immediately after surgery (postoperatively). The mean APTT was not significantly different between the two groups (Table 2). In the non-PEX group, the mean APTT was significantly higher postoperatively than at baseline (*p* = 0.008). In the PEX group, the mean APTT was not significantly different between baseline and postoperatively.

The mean PT-INR at baseline was not significantly different between the two groups. The mean postoperative PT-INR was significantly higher in the non-PEX than PEX group (*p* = 0.040) (Table 2). In the PEX group, the mean PT-INR was not significantly different between baseline and postoperatively. In the non-PEX group, the mean PT-INR was significantly higher postoperatively than at baseline (*p* < 0.0001).

The mean platelet count was significantly lower in the PEX than non-PEX group. A baseline platelet count of < 100 ×  10^3^/μl was found in 8% of patients in the PEX group. Neither group showed a significant difference in the mean platelet count between baseline and postoperatively.

The mean fibrinogen level was significantly lower in the PEX than non-PEX group (Table 2). In the PEX group, a fibrinogen level of < 150 mg/dl was found in 50% of patients at baseline and 12% of patients postoperatively. The mean number of PEX therapy was 2.85 ± 0.99 (Additional file 1). In the PEX group, the mean fibrinogen level was not significantly different between baseline and postoperatively. In the non-PEX group, the fibrinogen level was significantly lower postoperatively than at baseline.

### Comparison of ROTEM® parameters

**Fig. 1:**
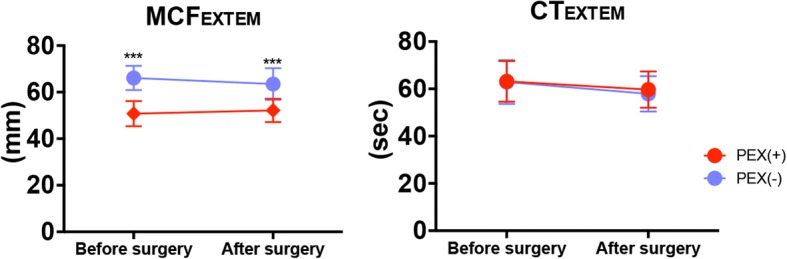
MCF and clotting time of EXTEM in the PEX group (red circles) and non-PEX group (blue circles). Data are presented as mean ± standard deviation. Sidak’s multiple-comparison post hoc test was utilized for two-way analysis of variance. ^***^p < 0.001

ROTEM® parameters were measured at baseline and postoperatively. The MCF_EXTEM_ was significantly lower in the PEX than non-PEX group (Fig. 1).

**Fig. 2:**
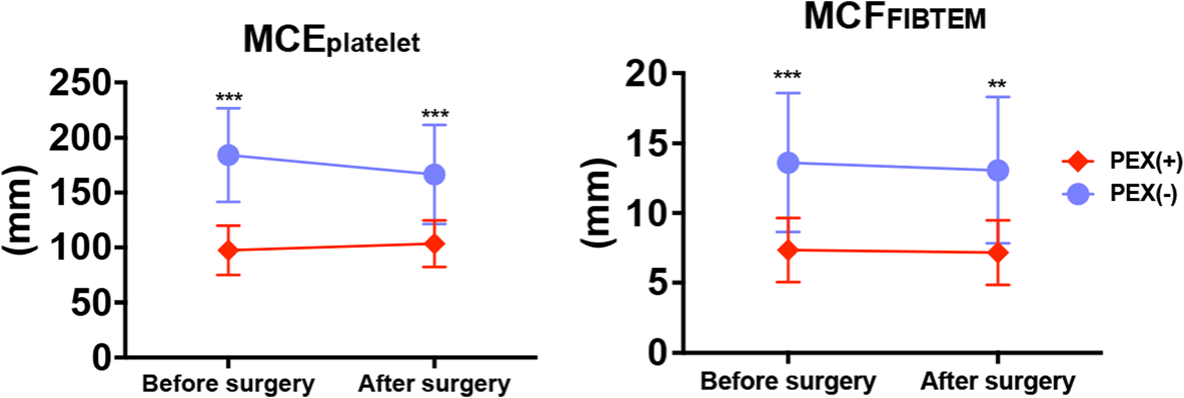
MCE of FIBTEM and the difference in the MCE between EXTEM and FIBTEM in the PEX group (*n* = 13, red circles) and non-PEX group (*n* = 15, blue circles). Data are presented as mean ± standard deviation. Sidak’s multiple-comparison post hoc test was utilized for two-way analysis of variance. ^**^p < 0.01, ^***^p < 0.001

In the PEX group, MCF_EXTEM_ values of < 50 mm were found in 50% of patients at baseline and 42% of patients postoperatively. There were no significant differences in the clotting time of EXTEM between the two groups (Fig. 1). MCF_FIBTEM_ and MCE_platelet_ were significantly lower in the PEX than non-PEX group (Fig. 2). MCF_FIBTEM_ values below reference range were found in 67% of patients in the PEX group.

### Fluid balance

The FFP transfusion volume during surgery was 1200 [1200] ml in the PEX group and 0 [0] ml in the non-PEX group (*p* < 0.0001). The 5% albumin transfusion volume during surgery was 1250 [1005] ml in the PEX group and 200 [350] ml in the non-PEX group (*p* = 0.03). The blood loss volume during surgery was 400 [546.5] g in the PEX group and 168 [98] g in the non-PEX group (*p* = 0.011). The blood loss volume during the intensive care unit (ICU) stay was 200 [271.5] g in the PEX group and 0 [95] g in the non-PEX group (*p* < 0.0001). No patient received platelet concentrate and cryoprecipitate during surgery.

### Correlation between ROTEM parameters and perioperative blood loss

In the PEX group, the bleeding volume during surgery was very strongly correlated with the baseline MCF_EXTEM_ and MCE_platelet_ but had no correlations with the baseline MCF_FIBTEM_, fibrinogen level, and platelet count. The blood loss volume during the ICU stay was strongly correlated with the postoperative MCF_EXTEM_ and MCE_platelet_ but had no correlations with the postoperative MCF_FIBTEM_, fibrinogen level, or platelet count (Table 3).

**Table 3:** Correlation between bleeding volume and ROTEM parameters in each group

	PEX (+)	PEX (−)	PEX (+)	PEX (−)
	During surgery		In ICU	
MCFEXTEM	−0.91 (< 0.001)	− 0.41 (0.126)	− 0.74 (0.008)	− 0.71 (0.004)
MCFFIBTEM	− 0.03 (0.942)	− 0.54 (0.039)	− 0.09 (0.776)	−0.51 (0.054)
Fibrinogen level	−0.23 (0.466)	−0.05 (0.892)	− 0.28 (0.939)	−0.26 (0.382)
Platelet level	−0.05 (0.886)	−0.54 (0.039)	0.05 (0.873)	−0.33 (0.232)
MCEplatelet	−0.91 (< 0.001)	− 0.40 (0.145)	− 0.76 (0.006)	−0.74 (0.003)
CTEXTEM	−0.009 (0.778)	0.12 (0.668)	0.28 (0.368)	0.31 (0.258)
alfaEXTEM	−0.45 (0.142)	−0.38 (0.162)	− 0.56 (0.063)	−0.51 (0.055)

Data are presented as *r* (*p*-value), where *r* is the correlation coefficient

In the non-PEX group, the bleeding volume during surgery had no correlations with the baseline MCF_EXTEM_, MCE_platelet_ and fibrinogen level, and had moderate correlations with MCF_FIBTEM_ and platelet count. The blood loss volume during the ICU stay had strong or moderate correlations with the postoperative MCF_EXTEM_, MCE_platelet_ and MCF_FIBTEM_,and no correlations with the postoperative fibrinogen level and platelet count (Table 3).

### Correlation between PEX sessions and clotting ability

In the PEX group, PEX therapy times had negligible correlation with the fibrinogen levels (*r* = 0.11, *p* = 0.71), MCE_platelet_ (*r* = 0.29, *p* = 0.36) and MCE_FIBTEM_ (*r* = 0.29, *p* = 0.36) before surgery (Table 4).

**Table 4:** Correlation between REX session times and clotting ability

Fibrinogen level	0.11 (0.71)
MCEplatelet	0.29 (0.36)
MCFFIBTEM	−0.23 (0.48)

Data are presented as *r* (*p*-value), where *r* is the correlation coefficient

## Discussion

In the present study, the bleeding volume during surgery and the ICU stay was significantly higher in the PEX than non-PEX group. In the PEX group, the bleeding volume during surgery and the ICU stay had a very strong or strong correlation with the MCF_EXTEM_ and MCE_platelet_. These results suggest that increased blood loss in the PEX group during surgery and the ICU stay was associated with decreased platelet function.

The plasma fibrinogen level significantly decreased after the performance of PEX with albumin replacement fluid, although there were no significant changes in the mean PT-INR and APTT. A previous study showed a prolonged PT and APTT and a reduced plasma fibrinogen level immediately after PEX performed with albumin replacement fluid because of significant loss of coagulation factors [3]. Recovery of PT and APTT takes 24 h, and recovery of fibrinogen takes about 72 h [18, 19]. In the present study, the last PEX session was performed until the day before previous surgery; thus, we consider that the mean PT-INR and APTT returned to baseline in the PEX group.

In the present study, the bleeding volume was significantly higher in the PEX than non-PEX group during both surgery and the ICU stay. There were no significant correlations between the bleeding volume and standard coagulation parameters. Standard coagulation parameters did not predict an increased bleeding volume in the PEX group, as previously described [20].

The clotting time in both groups was within the normal range. This suggests that the initial fibrin formation following thrombin generation was not disturbed in either group. The prolonged clot formation time and reduced alpha angle in the PEX group indicate that initial rate of fibrin polymerization was lower in the PEX than non-PEX group (Additional file 1). These results coincide with a previous study that examined the effects of PEX with albumin replacement fluid on hemostasis using ROTEM® [3].

Because PEX with albumin replacement fluid directly affects the blood coagulation system, there is concern that this treatment will increase the bleeding volume during surgery. The maximum clot firmness (MCF) is usually used to evaluate clot strength, but MCF does not reflect the actual physical properties of clot strength [14]. Unlike firmness, elasticity may be considered a reflection of the force with which the blood clot resists rotation within the device. It is important that the calculation of platelet component be performed using elasticity as opposed to clot firmness because of the nonlinear relationship between clot firmness and elasticity [21, 22]. Specifically, MCE reflects changes in platelet count. However, MCF sometimes remains unchanged in spite of increase in platelet count. Therefore, MCE is appropriate for calculating the platelet component of clot strength.

MCF_EXTEM_ and MCE_platelet_ were reduced in the PEX group of the present study. The bleeding volume during surgery and the ICU stay in this group had a very strong or strong correlation with MCF_EXTEM_ and MCE_platelet_. MCF_EXTEM_ represents the maximal viscoelastic strength of a clot. The MCF is associated with the fibrinogen concentration and platelet function and count [23]. Platelet counts of < 50,000/μL decrease the MCF_EXTEM_ [14]. Although the platelet counts were lower in the PEX than non-PEX group, all platelet counts in the PEX group were > 100 × 10^3^/μl. Furthermore, there was no significant correlation between the bleeding volume and platelet count in the PEX group.

MCE_platelet_, which indicates the difference in MCE between EXTEM and FIBTEM, reflects the whole blood platelet function [13–16, 24]. FIBTEM is influenced mainly by fibrinogen and factor XIII in a blood sample and by fibrin polymerization disorders. The reagent of FIBTEM contains a powerful platelet inhibitor; thus, FIBTEM indicates only fibrin clot formation. The difference in MCE between EXTEM and FIBTEM therefore indicates the contribution of platelet to the clot firmness [13–16, 24]. A very strong correlation was present between MCF_EXTEM_ and MCE_platelet_ in the PEX group; however, we observed no significant correlation between MCF_EXTEM_ and the fibrinogen level or MCF_FIBTEM_ in this group (Additional file 1). These results suggest that MCF_EXTEM_ was associated with MCE_platelet_ rather than the fibrinogen level in the PEX group. Previous study reported that platelet aggregation was significantly impaired during cardiopulmonary bypass (CPB) [25–28]. They supposed that direct contact of platelets with CPB circuit induces some changes in the expression of molecules involved in adhesion and aggregation or signaling pathway. However, it has not been clarified for the changes of platelet function after plasma exchange therapy. Then, further research is needed for this concern.

Despite almost patient during PEX series was administrated FFP in addition to albumin (Additional file 1), PEX performed with albumin replacement fluid resulted in lower preoperative fibrinogen levels in the PEX group and fibrinogen levels of < 150 mg/dl were observed in 50% of patients in the PEX group. The prolonged clot formation time and reduced clot firmness in the PEX group indicated abnormal clot formation (Additional file 1). The reduced fibrinogen level might have been responsible for the prolonged clot formation time and reduced clot firmness in the PEX group.

MCF_FIBTEM_ is correlated with the plasma fibrinogen level during surgery and the ICU stay. MCF_FIBTEM_ was lower in the PEX than non-PEX group. A low plasma fibrinogen level has been shown to be a risk factor for perioperative bleeding [29]. However, we found no significant correlation between the bleeding volume and MCF_FIBTEM_ or fibrinogen level during surgery and the ICU stay. These results suggest that decreased platelet function rather than fibrin-based clot firmness might have been responsible for the bleeding volume during surgery and the ICU stay in the PEX group.

## Conclusions

In the present study, the bleeding volume was higher in the PEX than non-PEX group. This might have been mainly because of the low platelet level and function caused by PEX. Thus, the difference in MCE between EXTEM and FIBTEM might be a more reliable index of coagulability than fibrinogen levels in patients undergoing ABO-incompatible LDKT with PEX performed with albumin replacement fluid. We should consider administration of platelet components in patients undergoing ABO-incompatible LDKT with PEX when the difference in clot strength between EXTEM and FIBTEM is decreased in spite of normal platelet counts.

## Additional file

Additional file 1: **Table S1.** Breakdown of replaced plasma volume during PEX. **Table S2.** Correlation between bleeding volume and ROTEM parameters. **Table S3.** Correlation between MCFEXTEM and ROTEM parameters. (DOCX 25 kb)

## Abbreviations

APTT: activated partial thromboplastin time
FFP: fresh frozen plasma
ICU: intensive care unit
LDKT: living-donor kidney transplantation
MCE: maximum clot elasticity
MCF: maximum clot firmness
PEX: plasma exchange therapy
PT: prothrombin time
ROTEM: rotational thromboelastometry
