# Supplementary material for: Effects of preoperative plasma exchange therapy with albumin replacement fluid on blood coagulation in patients undergoing ABO-incompatible living-donor kidney transplantation using rotational thromboelastometry
Source: BMC Anesthesiol. 2018 Jun 19;18:68. doi: 10.1186/s12871-018-0536-2 (PMC6008919; doi:10.1186/s12871-018-0536-2)
Supplement: Supplementary file 1 — Table S1. Breakdown of replaced plasma volume during PEX. Table S2. Correlation between bleeding volume and ROTEM parameters. Table S3. Correlation between MCFEXTEM and ROTEM parameters. (DOCX 25 kb) [file 12871_2018_536_MOESM1_ESM.docx]

Table S1 Breakdown of replaced plasma volume during PEX

| patients number | replaced plasma (ml) | Amount of  Albumin (ml) | Amount of  FFP (ml) | PEX times |
| --- | --- | --- | --- | --- |
| 1 | 1540 | 1500 | 0 |  |
|  | 1580 | 1500 | 0 |  |
|  | 1790 | 750 | 960 | 3 |
| 2 | 1580 | 1500 | 0 |  |
|  | 1830 | 750 | 960 |  |
|  | 1700 | 1500 | 0 |  |
|  | 1820 | 750 | 960 |  |
|  | 1810 | 750 | 960 | 5 |
| 3 | 1456 | 1500 | 0 |  |
|  | 1473 | 1500 | 0 |  |
|  | 1804 | 0 | 1920 | 3 |
| 4 | 1600 | 1500 | 0 | 1 |
| 5 | 1580 | 1500 | 0 |  |
|  | 1600 | 1500 | 0 |  |
|  | 1800 | 750 | 960 |  |
|  | 1830 | 750 | 960 | 4 |
| 6 | 1660 | 1500 | 0 |  |
|  | 1870 | 750 | 960 |  |
|  | 1820 | 750 | 960 | 3 |
| 7 | 1469 | 1500 | 0 |  |
|  | 1388 | 1500 | 0 | 2 |
| 8 | 1495 | 1500 | 0 |  |
|  | 1489 | 1500 | 0 |  |
|  | 1513 | 750 | 960 | 3 |
| 9 | 1800 | 750 | 960 |  |
|  | 1850 | 750 | 960 | 2 |
| 10 | 1515 | 1500 | 0 |  |
|  | 1521 | 1500 | 0 |  |
|  | 1546 | 1500 | 0 | 3 |
| 11 | 1488 | 1500 | 0 |  |
|  | 1471 | 1500 | 0 |  |
|  | 1192 | 750 | 960 | 3 |
| 12 | 1580 | 1500 | 0 |  |
|  | 1840 | 750 | 960 | 2 |
| 13 | 1590 | 1500 | 0 |  |
|  | 1890 | 750 | 960 |  |
|  | 1830 | 750 | 960 | 3 |

Tables S2 Correlation between bleeding volume and ROTEM parameters

|  | PEX (+) | PEX (−) | p value |
| --- | --- | --- | --- |
| MCFEXTEM at baseline (mm) | 50.8 ± 5.4 | 66.1 ± 5.2 | <0.001 |
| MCFEXTEM postoperatively (mm) | 52.2 ± 5.0 | 63.5 ± 6.9 | <0.001 |
| AlphaEXTEM at baseline (degrees) | 65.6 ± 4.2 | 78.1 ± 3.1 | <0.001 |
| AlphaEXTEM postoperatively (degrees) | 65.0 ± 4.8 | 74.6 ± 5.4 | <0.001 |
| CFTEXTEM at baseline (s) | 126.5 ± 28.6 | 61.5 ± 15.1 | <0.001 |
| CFTEXTEM postoperatively (s) | 130.7 ± 27.2 | 79.0 ± 28.1 | <0.001 |

Table S3 Correlation between MCFEXTEM and ROTEM parameters

|  |  | PEX (+) | PEX (−) | PEX (+) | PEX (−) |
| --- | --- | --- | --- | --- | --- |
|  |  | During surgery | | In ICU | |
| Fibrinogen level |  | 0.25  (0.422) | 0.82  (0.006) | 0.08  (0.812) | 0.49  (0.091) |
| MCEplatelet |  | 0.99  (<0.001) | 0.99  (<0.001) | 0.99  (<0.001) | 0.99  (<0.001) |
| MCFFIBTEM |  | 0.28  (0.367) | 0.80  (<0.001) | 0.39  (0.209) | 0.31  (0.261) |
